# Comparison of ovulation induction with letrozole plus dexamethasone and letrozole alone in infertile women with polycystic ovarian disease: An RCT

**DOI:** 10.18502/ijrm.v13i4.6893

**Published:** 2020-04-30

**Authors:** Farahnaz Farzaneh, Fatemeh Afshar

**Affiliations:** ^1^Infectious Disease and Tropical Medicine Research Center, Zahedan University of Medical Science, Zahedan, Iran.; ^2^Zahedan University of Medical Science, Zahedan, Iran.

**Keywords:** Letrozole, Dexamethasone, PCOS, Induction ovulation.

## Abstract

**Background:**

Infertility is characterized by the inability to obtain a successful pregnancy after 6 months or more with unprotected and regular intercourse. In developing countries, the incidence of infertility is 2%. The causes of infertility could be male factor or female factor, or mixed factor.

**Objective:**

This study was conducted with the aim of comparison the ovarian response to letrozole alone and letrozole plus dexamethasone in infertile women with poly cystic ovarian disease (PCOS).

**Materials and Methods:**

This randomized clinical trial was conducted on 120 infertile women with PCOS referred to Ali-Ebne-Abitaleb hospital, Zahedan, Iran from February to August 2017 into two groups: group I received letrozole alone and group II recived letrozole plus dexamethasone. The endometrial thickness, follicle diameter, and ovulation were evaluated and compared by ultrasound on days 12 to 14.

**Results:**

The mean thickness of endometrium was not different between two groups. Pregnancy rate was 8% in letrozole group and 23% in Letrozole plus Dexamethasone (p = 0.024). Also, the mean diameter of follicles in two groups were not statistically significant.

**Conclusion:**

Overall, this study showed that dexamethasone may increase pregnancy rate.

## 1. Introduction

Infertility is non-pregnancy after one year or more of unprotected sexual intercourse (1, 2). according to a large systematic analysis (2012), approximately 45-52 million couples (on average 48.5 million couples) have this problem all over the world (2).

Female causes are the most common causes of infertility. Among these factors, ovulatory disorders are the most common causes of female infertility.

Induction ovulation is the main treatment for ovulation disorders, and letrozole, as the first-line treatment for induction ovulation, may be as effective as clomiphene. In different studies, the success rate of letrozole in induction ovulation was 70-84% and the pregnancy rate was reported to be 27-20% (3, 4).

The weak response of the ovary to the gonadotropins is one of the problems in assisted reproductive techniques, which occurs in 9-26% of cycles. The ovarian response to stimulation of gonadotropins is regulated by an insulin-like growth factor-1. Glucocorticoids can be indirectly associated with increasing in serum levels of growth hormone and IGF-1. Consequently, increasing of IGF-1 concentration in follicular fluid is effective in improving the responses of weak responders. Dexamethasone improves ovulation by reducing the effect of adrenal androgens on follicular growth (5-7).

The aim of this study was to evaluate and compare the response of ovaries to letrozole alone and dexamethasone with letrozole in infertile women with poly cystic ovarian syndrome (PCOS).

## 2. Materials and Methods

**Figure 1:**
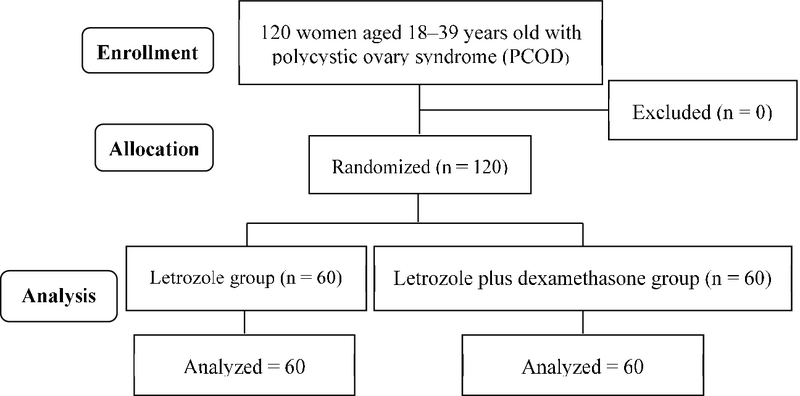
The COSORT flowchart of the study (letrozole vs letrozole plus dexamethasone).

This randomized clinical trial was conducted on 120 infertile women with PCOS, aged between 18-39 years, referred to infertility clinic of Ali-Ebne-Abitaleb hospital, Zahedan, Iran from February to August 2017 into two groups (n = 60/each): group I received letrozole alone and group II recived letrozole plus dexamethasone (Figure 1). The study was done with the block randomization method with 10 blocks and no blinding was performed.

The inclusion criteria were women with PCOS diagnosis and primary infertility, normal semen analysis of husband, and age between 18 and 39 years. Our exclusion criteria were co-administration of corticosteroid, endocrine disorders (such as thyroid disorders, high prolactin due to prolactinoma), and the history of ovarian surgery.

Letrozole (Iran Hormon company, Iran) was given in each group at the dose of 5 mg/day from the third to seventh day of the menstrual cycle for 5 days, and (Zahravi Pharma Co., Iran) was given at a dose of 0.5 mg / day with letrozole from day 4 to 15 orally. To determine the thickness of the endometrium, follicular diameter, and the evidence of ovulation, a vaginal ultrasound scan was performed on day 12-14 by an infertility fellowship.

### Ethical consideration

Written Informed consent was obtained from all participants in this study. The study has been approved by Zahedan University of Medical Sciences ethical committee (code: IR.ZAUMS.REC.1396.343)

### Statistical analysis

Data were analyzed using SPSS software (Statistical Package for the Social Sciences), version 22, and Chi-square and Independent *t* tests. P < 0.05 was considered statistically significant.

## 3. Results

The results of this study showed that the mean thickness of endometrium in the two groups was not statistically significant. Based on Chi-square test, the frequency of pregnancy in the letrozole group plus dexamethasone was significantly higher than letrozole alone group (23% vs. 8%, respectively; p = 0.024). Also, the mean diameter of follicles in two groups were not statistically significant (Table I).

**Table 1:** Comparison of mean endometrial thickness, diameter of follicles, and pregnancy rate in two study groups (n = 60/each)


**Variables**	**Group I (Letrozole)**	**Group II (Letrozole + Dexametasone)**	**P-value**
Endometrial thickness (mm)**	7.4 ± 2.2	8 ± 2.3	0.11${}^{a}$
Pregnancy rate (%)**	5 (%8)	14 (%23)	0.024${}^{b}$
Diameter of follicles (Right ovary) (mm)*	16.4 ± 3.3	16.9 ± 3.3	0.465${}^{a}$
Diameter of follicles (Left ovary) (mm)*	16.2 ± 3	16.2 ± 3	0.821${}^{a}$
* Data presented as Mean ± SD, ** Data presented as n(%), a: Independent *t* test, b: Chi-square			

## 4. Discussion

The aim of this study was to investigate and compare the response of ovaries to letrozole plus dexamethasone with letrozole alone in infertile women with PCOS. The results showed that the prevalence of pregnancy in dexamethasone plus letrozole group was significantly higher than letrozole alone. However, the thickness of the endometrium did not differ significantly between the two groups. Farzaneh and co-workers showed the degree of ovulation and also the pregnancy rate in the letrozole group was significantly higher than the clomiphene citrate group (2, 3).

A study by Al-Shaikh and colleagues examined the effect of prednisolone with clomiphene citrate on induction of ovulation in women with PCOS resistant to clomiphene citrate. In this study, the mean thickness of the endometrium as well as ovulation rate and frequency of fertility in the group receiving prednisolone plus clomiphene citrate was significantly higher than clomiphene citrate alone (5). Another study by Begum and others in Bangladesh (2012) examined the effect of dexamethasone plus letrozole in women with PCOS for infertility treatment. In this study 470 women were prescribed letrozole with doses 5 mg / day for 5 days that 280 women who did not respond to letrozole treatment were treated with letrozole plus dexamethasone (0.5 mg/day between the second and the 10th day) in the next cycle. Their results showed that 65% of women receiving dexamethasone, had ovulation signs in trans vaginal sonography in this cycle and 33% were pregnant (6). that conducted by Ashrafi and co-workers in Tehran (2014) to evaluate the effect of dexamethasone on ovarian response in 72 women over 35 years old in IVF cycle, reported that the mean count of oocytes in the dexamethasone group and the placebo group, was not significant. Also, the percentage of embryos and the mean count of transferred embryos in the two groups was not statistically significantl, but the number of human menopausal gonadotropin injections in the dexamethasone group was significantly low (7). Another study by Shabana and others in Egypt in 2017 examined and compared the effects of letrozole plus dexamethasone vs clomiphene citrate with dexamethasone in 60 women with PCOS (30 in each group) and reported that the mean thickness of the endometrium and also the mean diameter of the follicles in the letrozole group with dexamethasone were significantly higher than those receiving clomiphene citrate plus dexamethasone (8). Comparing the results of various studies in this field, we conclude that the use of dexamethasone increases ovarian responsiveness to ovulation-inducing drugs and also increases the fertility rate.

##  Conflict of Interest

The authors declare no conflict of interest regarding the publication of this paper.
